# Dual Induction of New Microbial Secondary Metabolites by Fungal Bacterial Co-cultivation

**DOI:** 10.3389/fmicb.2017.01284

**Published:** 2017-07-11

**Authors:** Jennifer Wakefield, Hossam M. Hassan, Marcel Jaspars, Rainer Ebel, Mostafa E. Rateb

**Affiliations:** ^1^Marine Biodiscovery Centre, Department of Chemistry, University of Aberdeen Aberdeen, United Kingdom; ^2^Pharmacognosy Department, Faculty of Pharmacy, Beni-Suef University Beni Suef, Egypt; ^3^School of Science and Sport, University of the West of Scotland Paisley, United Kingdom

**Keywords:** microbial co-cultivation, *Aspergillus fumigatus*, *Streptomyces leeuwenhoekii*, pseurotin G, luteoride D, brevianamide X

## Abstract

The frequent re-isolation of known compounds is one of the major challenges in drug discovery. Many biosynthetic genes are not expressed under standard culture conditions, thus limiting the chemical diversity of microbial compounds that can be obtained through fermentation. On the other hand, the competition during co-cultivation of two or more different microorganisms in most cases leads to an enhanced production of constitutively present compounds or an accumulation of cryptic compounds that are not detected in axenic cultures of the producing strain under different fermentation conditions. Herein, we report the dual induction of newly detected bacterial and fungal metabolites by the co-cultivation of the marine-derived fungal isolate *Aspergillus fumigatus* MR2012 and two hyper-arid desert bacterial isolates *Streptomyces leeuwenhoekii* strain C34 and strain C58. Co-cultivation of the fungal isolate MR2012 with the bacterial strain C34 led to the production of luteoride D, a new luteoride derivative and pseurotin G, a new pseurotin derivative in addition to the production of terezine D and 11-*O*-methylpseurotin A which were not traced before from this fungal strain under different fermentation conditions. In addition to the previously detected metabolites in strain C34, the lasso peptide chaxapeptin was isolated under co-culture conditions. The gene cluster for the latter compound had been identified through genome scanning, but it had never been detected before in the axenic culture of strain C34. Furthermore, when the fungus MR2012 was co-cultivated with the bacterial strain C58, the main producer of chaxapeptin, the titre of this metabolite was doubled, while additionally the bacterial metabolite pentalenic acid was detected and isolated for the first time from this strain, whereas the major fungal metabolites that were produced under axenic culture were suppressed. Finally, fermentation of the MR2012 by itself led to the isolation of the new diketopiperazine metabolite named brevianamide X.

## Introduction

Natural products are considered as specialized metabolites that often appear to play no part in the primary metabolism of the producing organism but instead are thought to confer an evolutionary advantage under specific environmental conditions (Challis and Hopwood, 2003). They occupy a diverse chemical structural space that is unmatched by synthetic compounds and remain an eminent source for new drug leads. Of the 1562 new chemical entities (NCEs) which were approved by the FDA covering all diseases/sources in the time frame spanning January 1981 until December 2014, about 60% are natural products, their analogs, or mimics (Newman and Cragg, 2016). Additionally, there is a significant number of natural product or natural product-derived drugs currently in development pipeline. For example, between 2008 and 2013, approximately 100 drug candidates based on natural products were in various phases of clinical trials, or in the final stage of registration (Butler et al., 2014).

Microorganisms of both terrestrial and marine origin have a long track record as important sources of novel bioactive natural products. However, the frequent re-isolation of known compounds is one of the major challenges in the process of the discovery of new natural products. Recent advances in microbial genomics have unequivocally demonstrated that the biosynthetic potential of microbes for producing natural products is much higher than currently appreciated (Harvey et al., 2015), but it is commonly believed that a significant number of microbial gene clusters may be silent under standard laboratory fermentation conditions.

In the last decade, several methods have been developed to eventually activate these cryptic biosynthetic pathways and hence, to elicit the production of hitherto unexpressed chemical diversity. Genetic engineering (Bergmann et al., 2007), epigenetic modifiers (Nützmann et al., 2011), and the OSMAC approach (Bode et al., 2002) are currently the most commonly applied strategies to increase chemical diversity in microorganisms through manipulation or activation of biosynthetic genes (Hertweck, 2009; Luo et al., 2013; Bertrand et al., 2014). In addition to this, microbial co-cultivation (also called mixed fermentation), involving the cultivation of two or more microorganisms in the same confined environment, has also successfully been used for the induction of the expression of otherwise cryptic pathways, leading to the production of new microbial natural products. Co-cultivation can be considered an experimental imitation of the competition within natural microbe communities at a laboratory scale which could encourage the production of secondary metabolites, for example *via* signaling molecules (auto-regulator/quorum sensing molecules, siderophores, etc.) in their environment (Bertrand et al., 2014; Marmann et al., 2014). Alternative interpretations suggest that this effect could be related to the production of enzymes that activate the metabolite precursor produced by the producer strain, yielding the active metabolite, or that the inducer strain may induce epigenetic modifications in the producer strain (Abdelmohsen et al., 2015). Even though the precise mechanism behind these microbial interactions may thus not be clear, the advantage of the co-cultivation methodology is that prior knowledge of the signaling mechanism is not required. However, it is worth noting that in many cases, direct contact was necessary between bacteria/fungi to observe such effects (Scherlach and Hertweck, 2009).

There are a number of recent studies which have demonstrated that co-cultivation is a remarkably successful approach for the discovery of new bioactive natural products (Onaka et al., 2011; Rateb et al., 2013; Dashti et al., 2014; Ebrahim et al., 2016), and an overview is provided by Marmann et al. (2014). Moreover, microbial co-cultivation has been shown to increase the titre of a specific metabolite in some cases, for example for the yew tree fungal endophyte *Paraconiothyrium*, responsible for production of the potent anticancer drug paclitaxel. The concentration of this alkaloid was raised to approximately eightfold when the producer strain was co-cultivated with other fungal community members such as the bark-derived fungi *Alternaria* sp. or *Phomopsis* sp. (Soliman and Raizada, 2013). Recently, our group has demonstrated the efficiency of the co-culture approach when investigating hyper-arid desert bacterial isolates (Rateb et al., 2013). In the course of that previous study, when screening a series of fungal isolates, we identified strains of *Aspergillus fumigatus* to be particularly responsive to the presence of other microbes. These results prompted us to investigate further isolates of the same fungal species from other habitats. In the present study, we report on the co-cultivation of the marine-derived fungal isolate *A. fumigatus* MR2012, and our findings indicate for the first time the dual induction of newly detected bacterial and fungal metabolites that were not traced previously.

## Materials and Methods

### General Experimental Procedures

Optical rotations were recorded using a Perkin-Elmer 343 polarimeter. UV and IR spectra were measured on a Perkin-Elmer Lambda 25 UV-vis spectrometer and a Thermo Nicolet IR 100 FT-IR spectrometer, respectively. NMR data were acquired on a Varian VNMRS 600 MHz NMR spectrometer. High resolution mass spectrometric data were obtained using a Thermo LTQ Orbitrap coupled to an HPLC system (PDA detector, PDA autosampler, and pump). The following conditions were used: capillary voltage of 45 V, capillary temperature of 260°C, auxiliary gas flow rate of 10-20 arbitrary units, sheath gas flow rate of 40-50 arbitrary units, spray voltage of 4.5 kV, and mass range of 100-2000 amu (maximal resolution of 30000). For LC/MS, a C18 analytical HPLC column (5 μm, 4.6 mm × 150 mm) was used with a mobile phase of 0 to 100% MeOH over 30 min at a flow rate of 1 mL min^-1^. A Biotage Flash system (Part No: SP1-XOB1) Charlottesville, WA, United States was used for initial fractionation. Preparative HPLC separations were conducted using a C18 column (5 μm, 100 Å, 10 mm × 250 mm), connected to a binary pump, and monitored using a photodiode array detector.

### Microbial Strains

The marine fungal isolate MR2012 used in this study was isolated from a Red Sea sediment in Hurghada, Egypt in September 2011, and taxonomically identified on a molecular basis as *A. fumigatus* (El-Gendy and Rateb, 2015). The two bacterial isolates C34 and C58 were collected from the hyper-arid soil of Laguna de Chaxa, Salar de Atacama, Chile and identified as *Streptomyces leeuwenhoekii* subspecies C34 and C58, respectively (Okoro et al., 2009).

### Fermentation, Extraction, and Isolation

Three different media; ISP2 (Shirling and Gottlieb, 1966), GYE medium composed of (g/L) glucose 10, yeast extract 10, and F-medium (Tian et al., 2017) composed of (g/L) sucrose 100, glucose 10, casamino acids 0.1, yeast extract 5, MOPS (3-*N*-morpholinopropanesulfonic acid) 21, K_2_SO_4_ 0.25 × 10^-6^, MgCl_2_ 6H_2_O 1.0 × 10^-6^, were used for small scale fermentation to screen for secondary metabolite production. The seed culture of each strain was prepared by inoculating 50 mL of liquid ISP2 medium with a single colony of the bacteria or a small piece (approximately 1 cm^2^) of agar containing fungal mycelia, respectively, and incubating for 3 days at 30°C with shaking at 180 rpm. Then, 2.5 mL of the primary culture was used to inoculate 250 mL of each of the three media. To adjust fermentation parameters and ensure reproducibility of secondary metabolite production before commencing large scale cultivation, screening of secondary metabolite profiles was conducted using LC-HRESIMS and LC-UV (in duplicate). The growth of both bacterial and fungal mycelia in their axenic or co-cultures, respectively, was also checked microscopically at different time intervals during the fermentation process (data not shown).

For large scale production, the seed culture of each strain was prepared as described above. Then 200 mL primary seed culture of each of fungal and bacterial isolates were used to inoculate 4 L of ISP2 medium as production medium in duplicate. For co-culture experiments, inoculation of the primary fungal culture was started 2 days before bacterial inoculation. Then, incubation of the secondary culture was conducted for 8 days at 30°C with shaking at 180 rpm as before. At the end of the incubation period, 50 g/L Diaion HP-20 resin was added to the culture media and shaken for 6 h at 180 rpm, then cultures were centrifuged (3000 rpm for 20 min) where the residue composed of cell mass and resin were washed with distilled water twice and extracted with MeOH, and subjected to LC-HRESIMS analysis. This extract was fractionated successively with *n*-hexane (3 mL × 250 mL), CH_2_Cl_2_ (3 mL × 300 mL), and then EtOAc (3 mL × 250 mL). Each solvent fraction was evaporated *in vacuo* and subjected to LC-HRESIMS and ^1^H NMR analysis, which revealed that the CH_2_Cl_2_ fraction was the one of interest for the fungal isolate and both fungal bacterial co-culture experiments. This CH_2_Cl_2_ fraction for each of the three fermentations was loaded on Flash Biotage using a FLASH 65i cartridge, solvent methanol/water 0–100%, flow rate 60 mL/min over 20 min and UV collection wavelengths 225 and 254 nm to produce six fractions. All of these fractions were monitored by LC-HRESIMS.

For the pure fungal isolate MR2012, fraction 3 was subjected to Sephadex LH-20 column using CH_2_Cl_2_:MeOH 1:1 as a mobile phase to obtain three subfractions A–C. Further purification of fraction B on Agilent HPLC system using semi-preparative Sunfire C18 column (250 mm × 10 mm, 5 μm) with CH_3_CN:H_2_O 40–80% over 30 min with a 2 mL/min flow led to the isolation of 0.9 mg of **1**.

For the fungal isolate MR2012/bacterial isolate C34 co-culture experiment, fraction 3 was directly subjected to HPLC using the same column with CH_3_CN:H_2_O 25–60% over 30 min and 2 mL/min flow which led to the isolation of 1.2 mg of **2**. Additionally, the injection of fraction 5 on HPLC using the same column with CH_3_CN:H_2_O 40–90% over 30 min and 2 mL/min flow led to the isolation of 0.8 mg of **3**.

Brevianamide X **1**: white amorphous; [α]^25^_D_ -86.9 (*c* 0.18, MeOH); UV (MeOH) λ_max_, nm (log 𝜀) 225 (4.62), 278 (3.90), 285 (2.85), 295 (3.70); IR (KBr) ν_max_ (cm^-1^) 3275, 1698, 1275, 1126; ^1^H and ^13^C NMR data were described in **Table 1**; HRESIMS: *m/z* 350.1470 [M+Na]^+^ (calcd for C_18_H_21_O_3_N_3_, 350.1475).

**Table 1:** Summary of ^1^H (600 MHz) and ^13^C (150 MHz) NMR spectroscopic data for brevianamide X **1** and luteoride D **2** in DMSO at 298 K.

	Brevianamide X 1		Luteoride D 2	
Atom	δ_C_,^1^ mult.	δ_H_, mult. (*J* in Hz)	δ_C_,^1^ mult.	δ_H_, mult. (*J* in Hz)
1				6.60 (d, 1.6)
2	127.9, CH	7.28 (s)	80.4, CH	5.03 (d, 1.7)
3	110.0, C		74.8, C	
4	128.2, C		132.3, C	
5	118.8, CH	7.61 (d, 8.1)	122.0, CH	7.02 (d, 7.8)
6	119.0, CH	7.06 (t, 7.9)	117.9, CH	6.63 (t, 7.6)
7	121.3, CH	7.15 (t, 8.1)	125.2, CH	7.31 (d, 7.8)
8	109.8, CH	7.50 (d, 8.2)	118.8, C	
9	135.9, C		147.0, C	
10	25.3, CH_2_	3.24 (m), 3.08 (m)		
11	54.9, CH	4.33 (t, 4.9)	143.2, C	
12		7.86 (s)	29.9, CH_2_	3.16 (d, 15.7), 2.79 (d, 15.7)
13	168.7, C		155.0, C	
14	58.1, CH	4.07 (t, 8.6)	124.6, CH	6.69 (d, 16.0)
15			129.9, CH	6.80 (d, 16.1)
16	165.2, C		142.4, C	
17	44.4, CH_2_	3.40 (m), 3.27 (m)	116.4, CH_2_	5.11 (br s), 5.01 (br s)
18	21.5, CH_2_	1.70 (m), 1.65 (m)	18.5, CH_3_	1.94 (s)
19	27.5, CH_2_	2.00 (q), 1.41 (m)		
20	76.1, CH_2_	5.46 (s)		
OMe	55.0, CH_3_	3.14 (s)	52.6, CH_3_	3.61 (s)

*^1^Extracted from HSQC and HMBC.*

Luteoride D **2**: white amorphous; [α]^25^_D_ -36.3 (*c* 0.12, MeOH); UV (MeOH) λ_max_, nm (log 𝜀) 212 (4.12), 258 (3.83), 329 (3.64); IR (KBr) ν_max_ (cm^-1^) 3315, 2950, 1720, 1432, 1340, 1215, 1135; ^1^H and ^13^C NMR data were described in **Table 1**; HRESIMS: *m/z* 315.1338 [M+H]^+^ (calcd for C_17_H_18_N_2_O_4_, 315.1339).

Pseurotin G **3**: light yellow amorphous; [α]^25^_D_ -11.3 (*c* 0.14, MeOH); UV (MeOH) λ_max_, nm (log 𝜀) 213 (3.62), 259 (4.12), 285 (3.74); IR (KBr) ν_max_ (cm^-1^) 3242, 1712, 1681, 1605, 1124; ^1^H and ^13^C NMR data were described in **Table 1**; HRESIMS: *m/z* 550.2177 [M+H]^+^ (calcd for C_29_H_31_O_8_N_3_, 550.2184).

## Results

In our previous work on fungal-bacterial co-culture, it became evident that the addition of the bacterial isolate was the trigger to initiate the production of fungal secondary metabolites (Rateb et al., 2013). In the current study, we aimed to monitor the effects of both bacteria and fungi on the induction of microbial secondary metabolites when inoculated in the same culture vessel. Before conducting microbial co-culture experiments, the chemical profiles of each strain were separately investigated using the OSMAC approach to obtain a maximum number of compounds produced, using a variety of media and both low and high nutrient conditions. Once the metabolite profile for each of these three strains was confirmed, the co-cultivation experiment was conducted.

**FIGURE 1:**
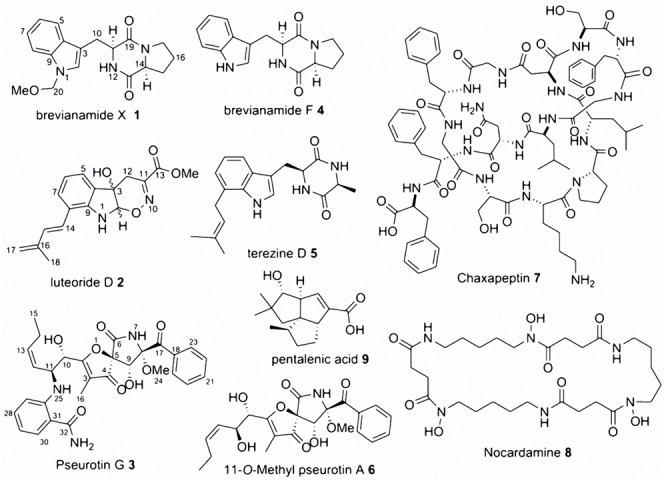
Compounds isolated from the microbial co-culture of *Aspergillus fumigatus* MR2012 and *Streptomyces leeuwenhoekii* strains C34 and C58 in addition to that isolated from the axenic marine-derived *A. fumigatus* MR2012.

The axenic marine-derived fungal isolate *A. fumigatus* MR2012 (El-Gendy and Rateb, 2015) was screened using three different media followed by media and mycelia extraction. Fractionation and multiple steps of SiO_2_ and Sephadex LH-20 followed by reversed phase semi-preparative HPLC purification led to the isolation and identification of a new metabolite belonging to the diketopiperazine family named brevianamide X **1** (**Figure 1**), in addition to the known metabolites brevianamide F **4**, cyclo(L-pro-L-val), cyclo(L-pro-L-ile), cyclo(L-pro-L-phe), cyclo(L-pro-L-leu), fumitremorgin C, spirotryprostatin A, 6-methoxy-spirotryprostatin C (El-Gendy and Rateb, 2015), pseurotin A (Rateb et al., 2013), *bis*(dethio)*bis*(methylthio)gliotoxin (Afiyatullov et al., 2005), and azaspirofurans A and B (Ren et al., 2010). These metabolites were identified based on comparing their spectral data with published data.

The molecular formula of **1** was established as C_18_H_21_O_3_N_3_ based on the HRESIMS analysis which gave an [M+Na]^+^ quasimolecular ion at *m/z* 350.1470. The analysis of ^1^H, ^13^C and multiplicity-edited HSQC NMR spectral data (**Table 1**) revealed the presence of two amide groups (δ_C_ 168.7, 165.2), a methoxy group (δ_C_ 55.0/δ_H_ 3.14), a methylene group attached to oxygen and nitrogen (δ_C_ 76.1/δ_H_ 5.46), a 1,2-disubstituted benzene ring (δ_H_ 7.61, 7.06, 7.15, 7.50), an olefinic singlet at δ_H_ 7.3, two α-protons at (δ_C_ 54.9/δ_H_ 4.33) and (δ_C_ 58.1/δ_H_ 4.07), four methylene groups, and an NH group at δ_H_ 7.86 (s).

**FIGURE 2:**
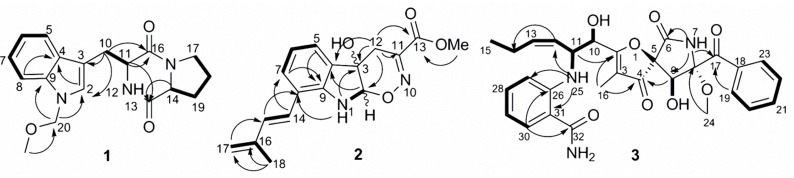
Key COSY correlations in bold and HMBC correlations shown with arrows of compounds **1**–**3**.

The COSY correlations of H-14 through H_2_-17 confirmed the proline ring substructure (**Figure 2**) and that of H-5 through to H-8 established the 1,2-disubstituted benzene ring, along with an olefinic singlet at δ_H_ 7.28 which indicated an indole moiety. This was confirmed by the HMBC correlations of H-2 to both C-4 and C-9, H-5 to C-3 and H-8 to C-4. The HMBC correlations of H_2_-10 to C-2, C-3, C-11 and C-19, H-14 to C-13 and H-11 to both C-13 and C-16 established the presence of an indolyl proline diketopiperazine skeleton which was identical to brevianamide F **4** structure (**Figure 2**) according to spectroscopic data comparison. The HMBC correlations of H_2_-20 to C-9, C-2 and C-21 were the key in the determination of the new substructure and its connection to the rest of the brevianamide F **4** structure (**Figure 2**). Since both **4** and **1** shared the same NOESY correlations, virtually identical ^13^C NMR spectral data and optical rotation values, and are assumed to be formed via a similar non-ribosomal peptide biosynthetic route, we proposed **1** to have the same absolute configuration as brevianamide F (Zhang et al., 2007). Hence, **1** was identified as a new marine fungal metabolite for which we propose the name brevianamide X.

**FIGURE 3:**
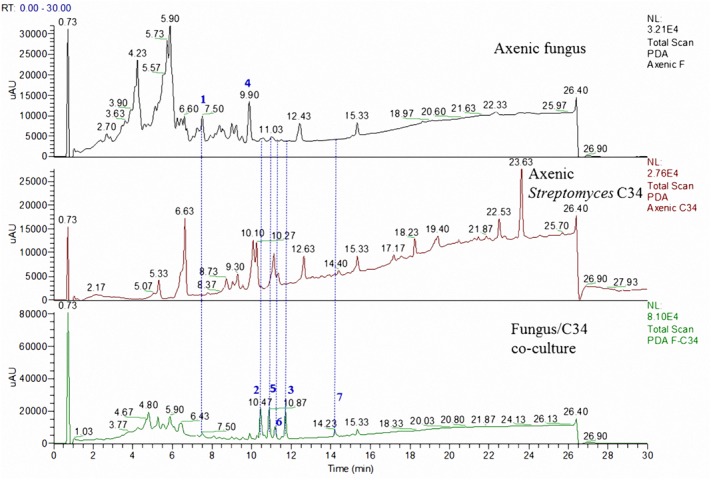
LC comparison of the microbial co-culture of *A. fumigatus* MR2012 and *S. leeuwenhoekii* strain C34 on ISP2 medium.

**FIGURE 4:**
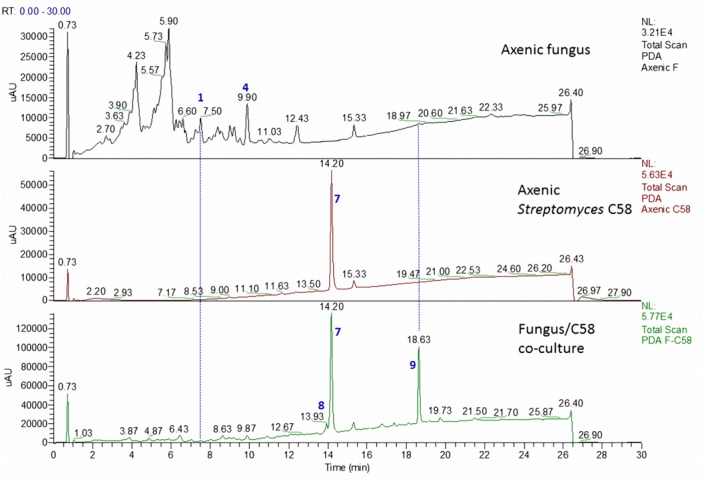
LC comparison of the microbial co-culture of *A. fumigatus* MR2012 and *S. leeuwenhoekii* strain C58 on ISP2 medium.

Our previous investigation of the Atacama Desert bacterial isolates *S. leeuwenhoekii* strains C34 and C58 led to the isolation of the ansamycin derivatives, chaxamycins (Rateb et al., 2011a), the macrolactin derivatives, chaxalactins in addition to the sedirophore nocardamine from *S. leeuwenhoekii* C34 (Rateb et al., 2011b), and the lasso peptide chaxapeptin from *S. leeuwenhoekii* C58 (Elsayed et al., 2015). All of these previously described compounds were identified in the current study using LC-HRESIMS analysis (**Figures 3**, **4**). As observed during our previous study, the profile pattern of *S. leeuwenhoekii* strain C34 changed markedly in the OSMAC approach (Rateb et al., 2011b), while it was fairly constant for *S. leeuwenhoekii* strain C58.

However, we observed a dramatic change in chemical profiles when each of the two bacteria was separately co-cultured together with the fungus as described in the experimental section. At this stage we carried out large scale co-cultivation, extraction and chromatographic separation, which was guided by focusing on unique peaks with retention times and UV spectra observed in the HPLC profiles which were not present in the profiles of either strain when cultivated on its own. **Table 2** represents all bacterial and fungal metabolites identified in axenic and co-culture flasks of these micro-organisms.

**Table 2:** Summary of the metabolites detected by LCMS in bacterial and fungal axenic culture and co-culture experiments.

Media	Strain C34			Strain C58			Fungus MR2012			Fungus/C34	Fungus/C58
Compounds	ISP2	GYE	F	ISP2	GYE	F	ISP2	GYE	F	ISP2	ISP2
Brevianamide F	-	-	-	-	-	-	+	-	+	-	-
Brevianamide X	-	-	-	-	-	-	-	-	+	-	-
Cyclo(L-pro-L-val)	-	-	-	-	-	-	+	+	-	-	+
Cyclo(L-pro-L-ile)	-	-	-	-	-	-	+	+	-	-	+
Cyclo(L-pro-L-phe)	-	-	-	-	-	-	+	+	-	-	+
Cyclo(L-pro-L-leu)	-	-	-	-	-	-	+	+	+	-	+
Fumitremorgin C	-	-	-	-	-	-	+	-	-	-	-
Spirotryprostatin C	-	-	-	-	-	-	+	-	-	-	-
Pseurotin A	-	-	-	-	-	-	+	+	+	-	-
B*is*(dethio)*bis*(methylthio)gliotoxin	-	-	-	-	-	-	+	-	+	-	-
Azaspirofuran A	-	-	-	-	-	-	+	-	+	-	-
Azaspirofuran B	-	-	-	-	-	-	+	-	+	-	-
Chaxamycins A–D	+	-	+	-	-	-	-	-	-	-	-
Chaxalactins A–C	+	-	-	-	-	-	-	-	-	-	-
Nocardamine	+	+	+	-	-	-	-	-	-	++	++
Chaxapeptin	-	-	-	+	+	+	-	-	-	+	++
Terezine D	-	-	-	-	-	-	-	-	-	+	-
Luteride D	-	-	-	-	-	-	-	-	-	+	-
Methylpseurotin A	-	-	-	-	-	-	-	-	-	+	-
Pseurotin G	-	-	-	-	-	-	-	-	-	+	-
Pentalenic acid	-	-	-	-	-	-	-	-	-	-	+

The microbial co-culture of *A. fumigatus* MR2012 and *S. leeuwenhoekii* strain C34 on ISP2 medium (**Figure 3**) led to the production of the bacterial metabolite chaxapeptin **7**, the gene cluster of which is known to be present in the bacterial strain. However, no chaxapeptin production was obtained when the bacterium was fermented using six different media and different cultivation parameters during previous studies (Rateb et al., 2011b; Gomez-Escribano et al., 2015). On the other hand, all other bacterial metabolites that were previously detected were not produced in the current co-culture experiment, except for nocardamine. Interestingly, the HPLC profile of the co-culture was dominated by fungal metabolites, including the known diketopiperazine terezine D **5**, the new luteoride derivative luteoride D **2**, the known 11-*O*-methylpseurotin A **6**, and a new pseurotin derivative, pseurotin G **3**. None of these compounds were observed when the fungus was cultivated on its own.

HRESIMS analysis afforded an [M+H]^+^ quasimolecular ion at *m/z* 315.1338, establishing the molecular formula of **2** as C_17_H_18_N_2_O_4_. The analysis of ^1^H, ^13^C and multiplicity-edited HSQC NMR spectral data (**Table 1**) indicated the presence of a methoxy group (δ_C_ 52.6/δ_H_ 3.61), a methyl group connected to a double bond (δ_C_ 18.5/δ_H_ 1.94), a methylene group (δ_C_ 29.9/δ_H_ 3.16, 2.79), a geminal olefinic methylene group (δ_C_ 116.4/δ_H_ 5.11, 5.01), a 1,2,3-trisubstituted benzene ring (δ_H_ 7.02, 6.63, 7.31), a hemiaminal (δ_C_ 80.4/δ_H_ 5.03), and a disubstituted *E*-olefinic moiety [δ_H_ 6.69 (d, 16.0) and 6.80 (d, 16.1)].

The COSY correlations of H-5 through H-7, 1-NH to H-2, and the HMBC correlations of 1-NH to C-8 and C-3, H-2 to C-9, C-3 and C-12, H-5 to C-3 and C-9 confirmed the presence of indoline ring substructure substituted at position 8 (**Figure 2**). The COSY correlations of H-14 to H-15, H_2_-17 to H_3_-18 and the HMBC correlations of H_3_-18 to C-15, C-16 and C-17, H-15 to C-8 and H-14 to C-7 indicated a 2-methylpenta-1,3-dienyl moiety connected to the indoline ring at position 8. The HMBC correlations of the methyl group at δ_H_ 3.61 to C-13 indicated a -COOCH_3_ group. The remaining two double bond equivalents as well as one nitrogen and one oxygen implied by the molecular formula, were attributed by the presence of an oxazine ring connected to the indoline moiety. This was confirmed by the HMBC correlations of H_2_-12 to C-2, C-3 and C-11, establishing a dihydro-[1,2]oxazino[6,5-*b*]indol-4a(4H)-ol moiety, and the connection of the -COOCH_3_ group was corroborated through the HMBC correlations of H_2_-12 to C-13. No NOEs for either H-2 or 3-OH were observed in DMSO-*d*_6_ or CD_3_OD, so the relative configuration at these two positions could not be determined. Thus, compound **2** was identified as a new natural product for which the name luteoride D is proposed. Although indoline moieties are frequently encountered in fungal natural products, to the best of our knowledge this is the first report of a oxazino[6,5-*b*]indole nucleus in nature. Based on inspection of its structure, **2** may be formed from the known luteoride A via nucleophilic attack of oxime OH moiety to the olefinic carbon of the indole ring. The latter compound is a prenylated tryptophan analog that was reported recently from the entomopathogenic fungus *Torrubiella luteorostrata* when induced by the histone deacetylase inhibitor suberoyl *bis*-hydroxamic acid (Asai et al., 2011).

The molecular formula C_29_H_31_O_8_N_3_ was established for compound **3** based on the HRESIMS a [M+H]^+^ quasimolecular ion at *m/z* 550.2177. The analysis of the ^1^H, ^13^C, and multiplicity-edited HSQC NMR data of **3** (**Table 3**) indicated the presence of a monosubstituted benzene ring (δ_H_ 8.27, 7.83, 7.54), a 1,2-disubstituted benzene ring (δ_H_ 7.96, 7.24, 6.65, 6.52), a disubstituted *Z* double bond (*J* = 10.9 Hz), one aliphatic and one allylic methyl group at δ_C/H_ 13.7/0.81 and 5.42/1.67, respectively, one O-methyl group at δ_C/H_ 51.4/3.23, four carbonyls including one α,β-unsaturated ketone at δ_C_ 196.5, one ketone at δ_C_ 196.1, and two amide groups at δ_C_ 171.2 and 171.9, two NH groups (δ_H_ 8.43 and 9.96), and a oxygenated tetrasubstituted olefinic bond (δ_C_ 185.8 and 111.5). Comparison with reported data (Wang et al., 2011) indicated that **3** belonged to the pseurotin family of compounds.

**Table 3:** Summary of ^1^H (600 MHz) and ^13^C (150 MHz) NMR spectroscopic data for pseurotin G **3** in DMSO at 298 K.

	Pseurotin G 3	
Atom	δ_C_,^1^ mult.	δ_H_, mult. (*J* in Hz)
2	185.8, C	
3	111.5, CH	
4	196.5, C	
5	90.9, C	
6	171.9, C	
8	92.4, C	
9	74.7, CH	4.50 (s)
10	70.6, CH	4.63 (s)
11	52.2, CH	4.50 (s)
12	134.6, CH	5.48 (m)
13	125.3, CH	5.30 (m)
14	20.5, CH_2_	2.03 (m), 1.94 (m)
15	13.7, CH_3_	0.81 (t, 7.4)
16	5.42, CH_3_	1.67 (s)
17	196.1, C	
18	133.3, C	
19/23	130.7, CH	8.27 (d, 7.9)
20/22	128.2, CH	7.54 (t, 7.8)
21	133.7, CH	7.68 (t, 7.3)
24	51.4, CH_3_	3.23 (s)
26	148.0, C	
27	111.4, C	6.65 (d, 8.9)
28	132.1, CH	7.24 (t, 7.6)
29	114.5, CH	6.52 (t, 7.6)
30	129.0, CH	7.56 (d, 7.6)
31	114.2, C	
32	171.2, C	
7-NH		9.96 (s)
25-NH		8.43 (d, 8.2)

*^1^Extracted from HSQC and HMBC.*

The COSY correlations (**Figure 2**) confirmed the location of the two OH groups, confirmed the identity of the two aromatic rings, and established the chain from H-10 through H_3_-15. The HMBC correlation from H-19/23 to C-17 confirmed the attachment of the mono-substituted benzene ring to the carbonyl C-17, while the di-substituted benzene ring was identified as part of an anthranilamide moiety on the basis of HMBC correlations of H-30 to C-32 and of NH-25 to both C-27 and C-31. The attachment of the latter to the pseurotin core at position 11 was confirmed through the COSY correlation of NH-25 to H-11 and HMBC correlation of NH-25 to C-12. Furthermore, the mono-substituted benzene ring was connected to the pyrrolidinone moiety through the carbonyl C-17 as evident from HMBC correlations of both NH-7 and H-9 to C-17. The HMBC correlation of H-9 to C-4 confirmed the spirocyclic system, while the furanone ring was established on the basis of correlation between H_3_-16 to C-2, C-3, and C-4. Since both pseurotin A and **3** share the same NOESY correlations, displayed virtually identical ^13^C spectral data and optical rotation values, we assume **3** to have the same absolute configuration as pseurotin A, and propose the name pseurotin G for this new metabolite. A putative biosynthetic route leading to **3** is shown in Supplementary Figure S14 based on the biosynthetic pathway of pseurotin A published by Tsunematsu et al. (2014).

The microbial co-culture of *A. fumigatus* MR2012 and *S. leeuwenhoekii* strain C58 on ISP2 medium (**Figure 4**) led to the production of four simple diketopiperazines assumed to be produced by the fungus, since they were also observed in the axenic culture of the fungus. However, the titre of bacterial metabolite chaxapeptin **7** was dramatically increased, and additionally, the bacterium was induced to produce the known bacterial sesquiterpene pentalenic acid **9**, which had previously been isolated from various *Streptomyces* spp. (Takamatus et al., 2011), and the known siderophore, nocardamine **8**. The latter two were not observed in single cultures of the bacterium under different fermentation conditions. Based on inspection of their structures, these three compounds clearly are bacterial metabolites, and additionally, the gene clusters responsible for their biosynthesis were confirmed to be present in the genome of *S. leeuwenhoekii* strain C58 (data not shown). All three bacterial compounds were identified based on their HRESIMS analysis and comparison with the previously reported NMR data (Rateb et al., 2011a; Elsayed et al., 2015).

## Discussion

In the past few years, various researchers have established microbial co-cultivation as a powerful tool for mimicking the natural microbial environment and enhancing the production of specific metabolites, or inducing the production of new secondary metabolites not previously observed in the independent strain cultures. Activation of cryptic biosynthetic genes in a second microorganism may be stimulated through microbial crosstalk and may be interpreted as a defense mechanism triggered in response to a chemical signal from the other microorganism (Pettit, 2009; Schroeckh et al., 2009). Ten years ago, the application of co-cultivation (mixed fermentation) was still in its infancy, probably due to the fear of lack of reproducibility (Pettit, 2009) but since then, various studies have demonstrated that this approach is capable of delivering reproducible metabolite patterns, provided relevant fermentation parameters are first established and then carefully maintained.

In the present study, small scale fermentation of axenic cultures of bacteria and fungi, and of their co-cultures was conducted at different conditions, and was carefully monitored by LC-HRESIMS, LC-UV and microscopic analysis (data not shown) to adjust fermentation parameters and ensure reproducibility of secondary metabolite production. Once an optimized set of fermentation parameters was established, a large scale co-culture experiment was conducted at a scale of 4 L for natural product isolation. Chemical profiles observed for small and large scale cultivation were highly comparable (data not shown). Our selection of *A. fumigatus* as the fungal component was established as a results of a broader screening of different fungal strains, which revealed this species to be particularly prone to respond by displaying modified metabolite profiles upon co-cultivation with other microorganisms (data not shown). During the preliminary chemical screening of various isolates of *A. fumigatus*, strain MR2012 which had been obtained from a Red Sea sediment sample caught our attention as it produced a new metabolite, brevianamide X **1**, which was only observed upon fermentation in F-medium, but not in two other culture media. This finding highlights the importance of varying culture conditions and of using a variety of fermentation media for screening (Kang et al., 2013).

To date, co-culture studies reported the effect of one microorganism to affect the metabolite profile of the second microorganism. Our current investigation not only showed that co-culture can induce the production of new metabolites, but highlighted the fact that this may work in both directions. Comparing the chemical profiles of the pure cultures to those from the co-culture, a great diversity was shown (**Figures 3**, **4**). In the microbial co-culture of *A. fumigatus* MR2012 and *S. leeuwenhoekii* C34, the bacterial strain appears to have suppressed the production of most of fungal metabolites detected in the axenic culture. However, it induced the production of two fungal prenylated indole metabolites which were not traced before in the fungus; the known terezine D and the new luteoride D **2** featuring an oxazino[6,5-*b*]indole nucleus that was not previously reported in nature. Additionally, the new compound, pseurotin G **3** was also induced in the fungus. Based on analysis of its structure, **3** is assumed to be of hybrid polyketide and non-ribosomal peptide origin as was demonstrated for pseurotin A (Tsunematsu et al., 2014). A putative biosynthetic route for **3** is provided in Supplementary Figure S14. It is interesting to note that fungi have been found to readily incorporate anthranilamide or anthranilic acid (Xin et al., 2007; Li et al., 2013). Thus, the origin of the respective substructure in **3** which so far has not been observed for other pseurotin derivatives, i.e., whether it is produced by the fungus itself or by the bacterium, or may be even incorporated from the culture medium, cannot be established at this point.

Surprisingly, in the co-cultivation experiments of *A. fumigatus* MR2012 and *S. leeuwenhoekii* C34, the fungus appears to have suppressed all ansamycin and macrolactin derivatives that previously were observed in axenic cultures of the bacterium (Rateb et al., 2011b), while the lasso peptide chaxapeptin was produced at significant levels. Whereas genome scanning had revealed the presence of the chaxapeptin biosynthetic gene cluster in *S. leeuwenhoekii* C34 (Gomez-Escribano et al., 2015), our previous chemical analysis had failed to detect the expression of this metabolite when the bacterium was fermented using six different media and a variety of cultivation parameters (Rateb et al., 2011b). It is worth noting that the production of antimicrobial lasso peptides could be triggered by competing nutrient scarcity in the culture vessel (Hegemann et al., 2015).

In the microbial co-culture of *A. fumigatus* MR2012 and *S. leeuwenhoekii* C58, the bacterial strain appears to have suppressed the production of the fungal metabolites that were present in the axenic culture. However, the bacterium was induced to produce pentalenic acid and nocardamine, both of which were not observed in the single bacterial culture under different fermentation conditions. Additionally, the titre of the lasso peptide chaxapeptin was greatly increased. It is worth noting that none of these metabolites proved to have antifungal effects when screened in previous studies.

Interestingly, siderophores such as nocardamine have been identified as auto-regulator/quorum sensing molecules (Bertrand et al., 2014). In order to assess whether the effects observed in the present study are due to some metabolites acting as signaling molecules, or may be explained in terms of mere antimicrobial effects, further studies need to be conducted.

In summary, co-cultivation is an ecologically driven approach which has become a powerful method to induce previously unexpressed biosynthetic pathways and increase the metabolic capacity of chemically prolific microorganisms beyond the boundaries that can be reached by routine axenic cultivation. In this current study, a bi-lateral cross talk that led to dual induction of both bacterial and fungal metabolites in the same culture flask was proved for the first time.

## Author Contributions

Conceived and designed the experiments: JW, HH, MJ, RE, and MR. Performed the experiments: JW, HH, and MR. Analyzed the data: JW, HH, MJ, RE, and MR. Contributed reagents/materials/analysis tools: JW, MJ, RE, and MR. Wrote and enriched the literature: JW, HH, MJ, RE, and MR.

## Conflict of Interest Statement

The authors declare that the research was conducted in the absence of any commercial or financial relationships that could be construed as a potential conflict of interest. The reviewer TS and handling Editor declared their shared affiliation, and the handling Editor states that the process nevertheless met the standards of a fair and objective review.
