# Supplementary material for: Dual Induction of New Microbial Secondary Metabolites by Fungal Bacterial Co-cultivation
Source: Front Microbiol. 2017 Jul 11;8:1284. doi: 10.3389/fmicb.2017.01284 (PMC5504103; doi:10.3389/fmicb.2017.01284)
Supplement: Supplementary file 1 [file Data_Sheet_1.PDF]

Supplementary data

Dual induction of new microbial secondary metabolites by fungal bacterial co-cultivation

Jennifer Wakefield,[1] Hossam M. Hassan,[2] Marcel Jaspars,[1] Rainer Ebel,[1] and Mostafa E. Rateb[3,*]

[1] *Marine Biodiscovery Centre, Department of Chemistry, University of Aberdeen, Scotland, AB24 3UE, UK.*

[2] *Pharmacognosy Department, Faculty of Pharmacy, Beni Suef University, Beni Suef 62514, Egypt.*

[3] *School of Science & Sport, University of the West of Scotland, Paisley PA1 2BE, UK.*

*Correspondence: mostafa.rateb@uws.ac.uk Tel. +441418483072

Table of content:

S1. HRESIMS analysis of brevianamide X **1**. 2

S2. ^{1}H NMR spectrum of brevianamide X **1**. 2

S3. HSQC spectrum of brevianamide X **1**. 3

S4. HMBC spectrum of brevianamide X **1**. 3

S5. HRESIMS analysis of luteoride D **2**. 4

S6. ^{1}H NMR spectrum of luteoride D **2**. 4

S7. ^{13}C NMR spectrum of luteoride D **2**. 5

S8. HSQC spectrum of luteoride D **2**. 5

S9. HMBC spectrum of luteoride D **2**. 6

S10. HRESIMS analysis of pseurotin G **3**. 6

S11. ^{1}H NMR spectrum of pseurotin G **3**. 7

S12. HSQC spectrum of pseurotin G **3**. 7

S13. HMBC spectrum of pseurotin G **3**. 8

S14. Proposed biosynthetic pathway of pseurotin G **3**. 9

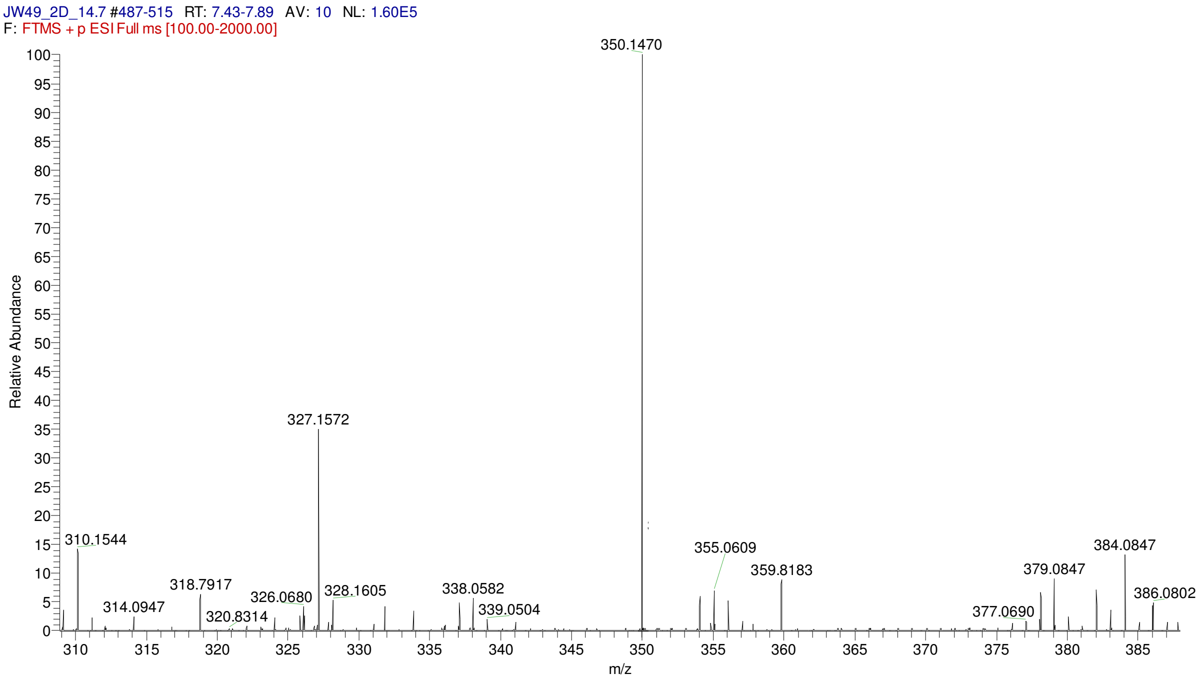

S1. HRESIMS analysis of brevianamide X **1**.

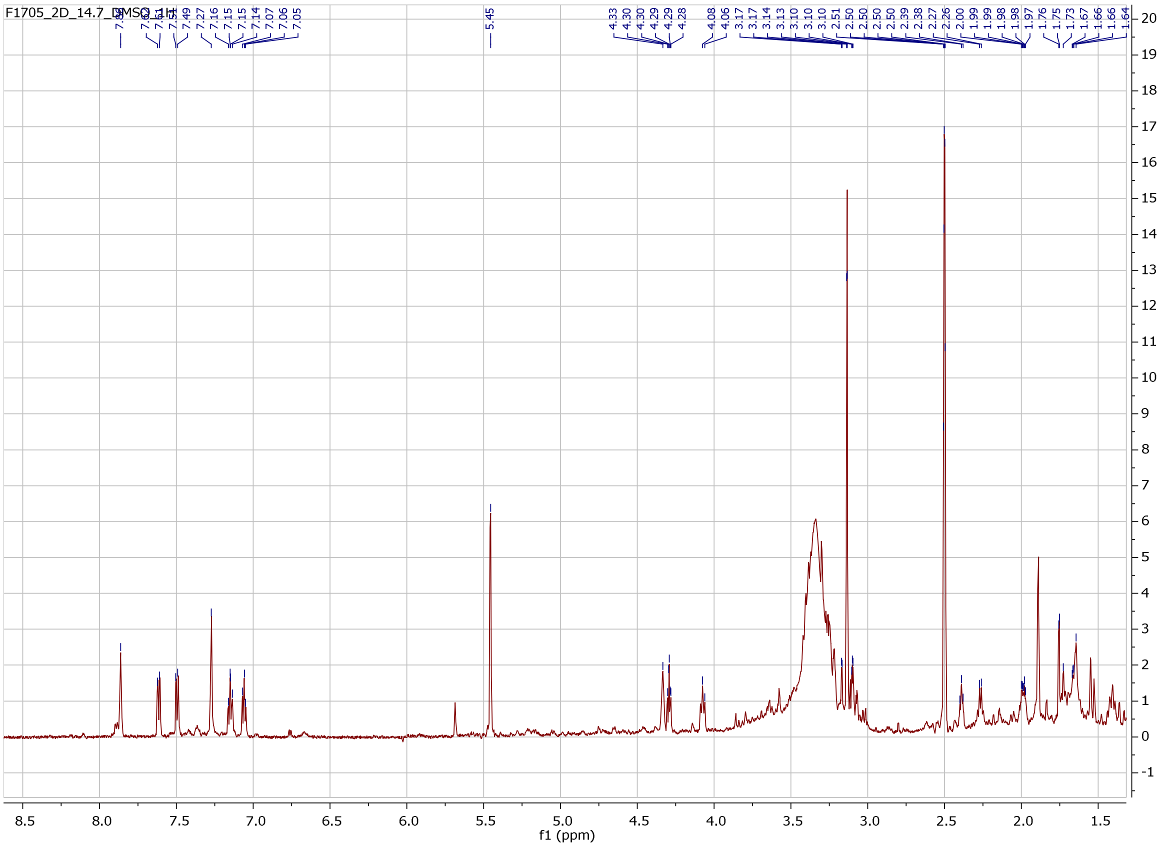

S2. ^{1}H NMR spectrum of brevianamide X **1**.

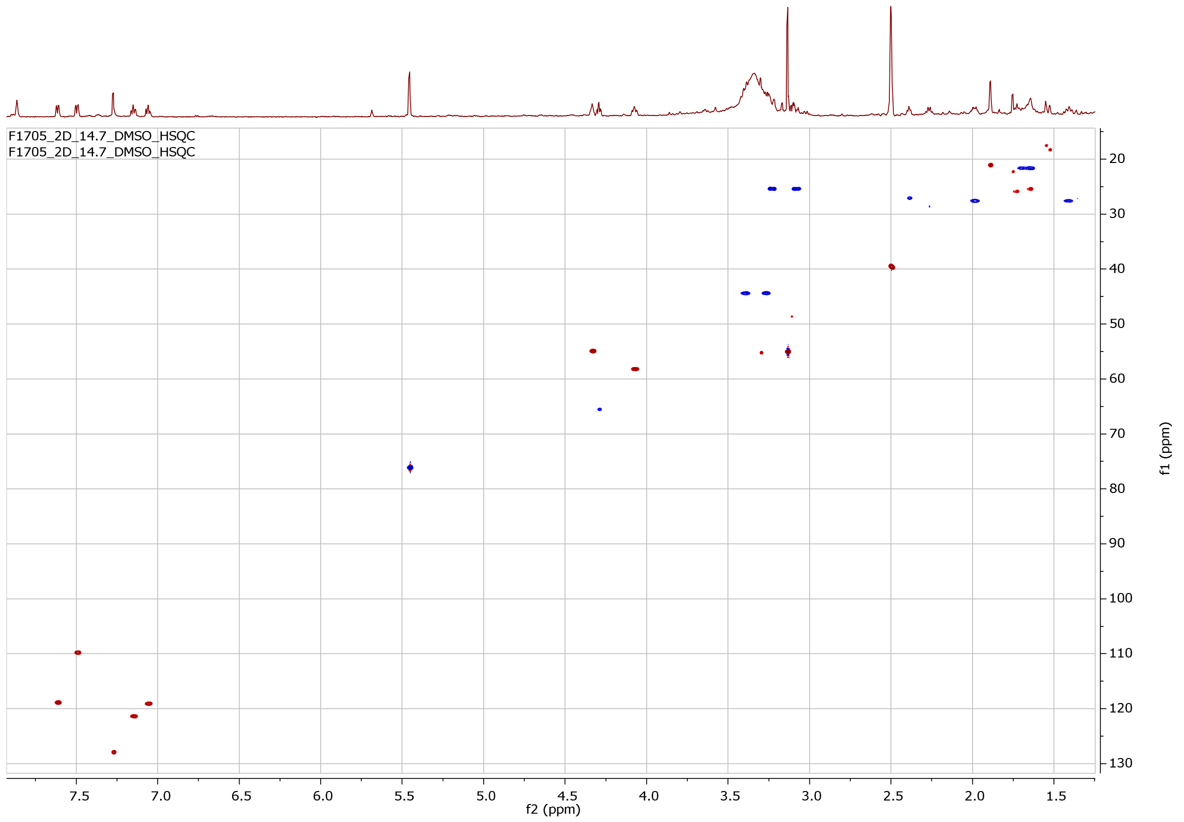

S3. HSQC spectrum of brevianamide X **1**.

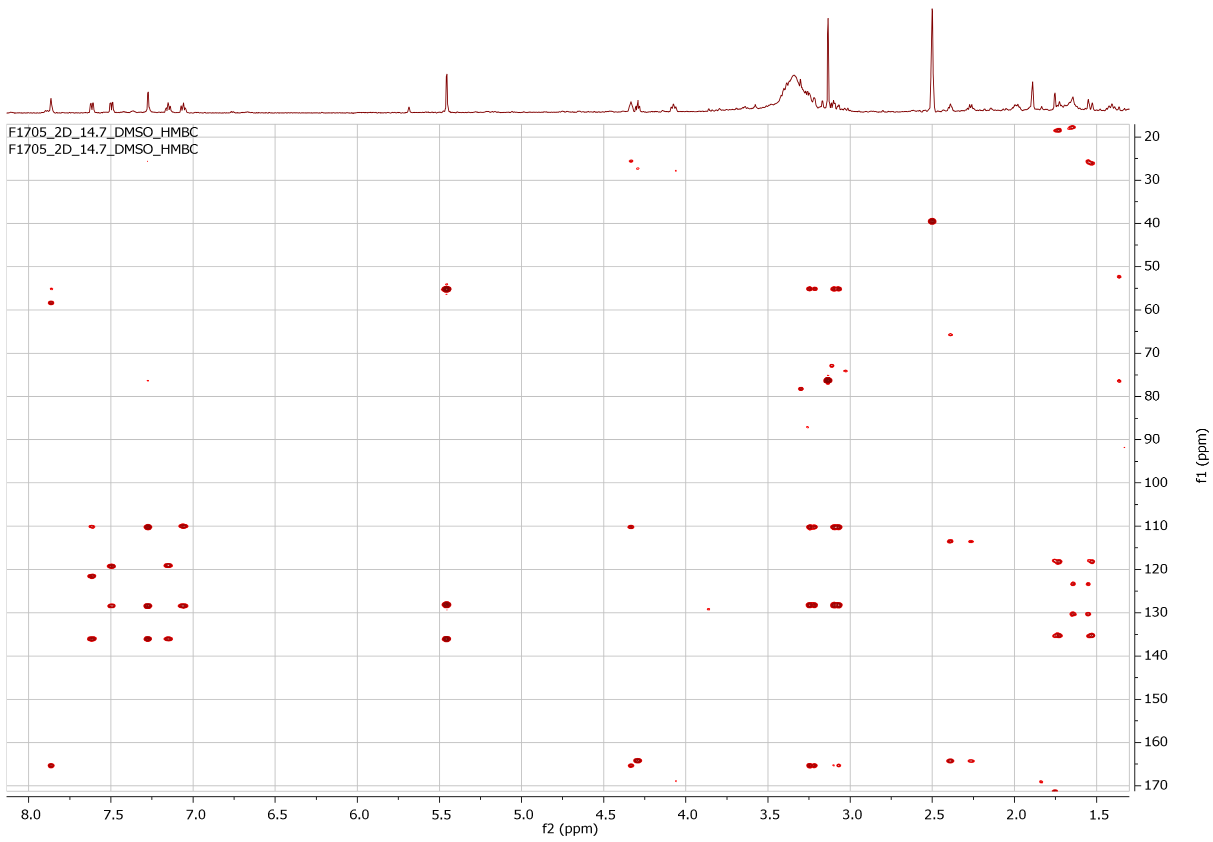

S4. HMBC spectrum of brevianamide X **1**.

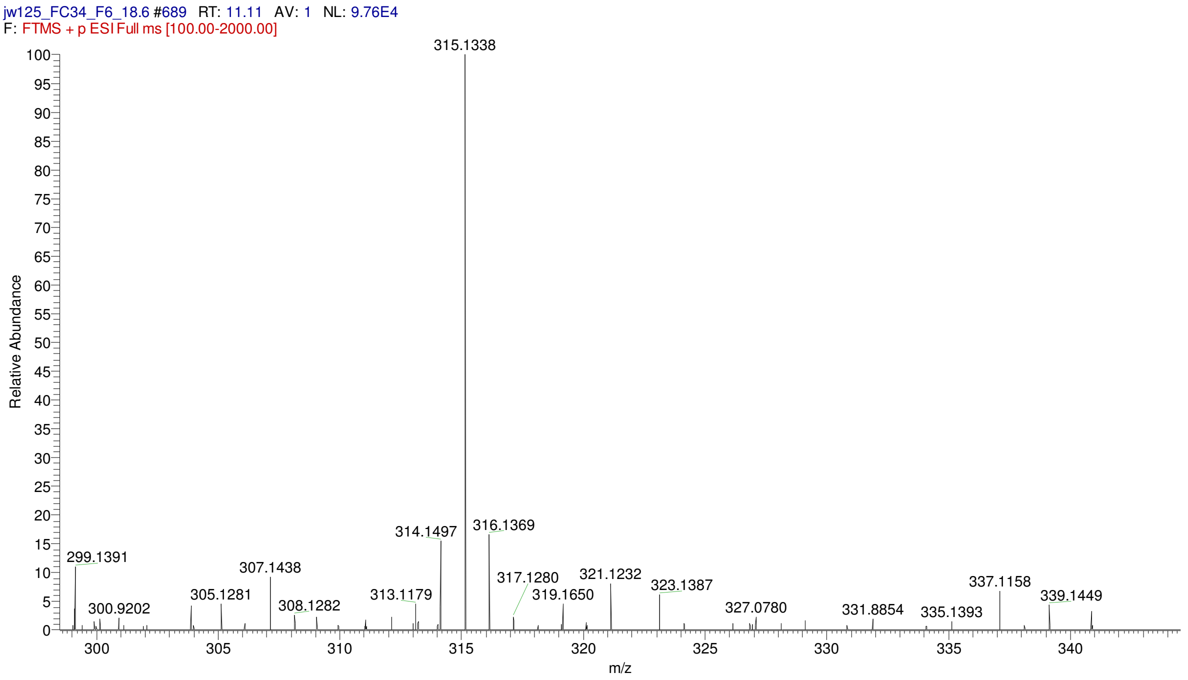

S5. HRESIMS analysis of luteoride D **2**.

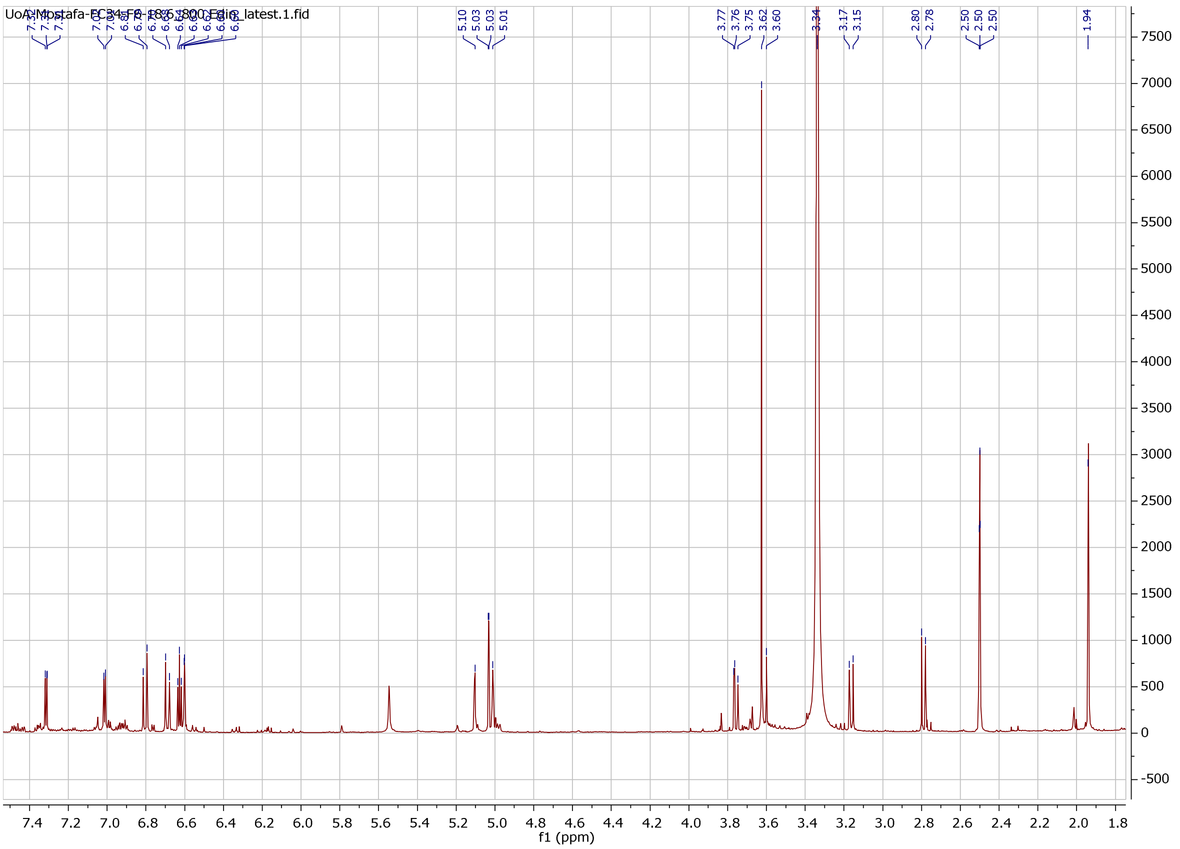

S6. ^{1}H NMR spectrum of luteoride D **2**.

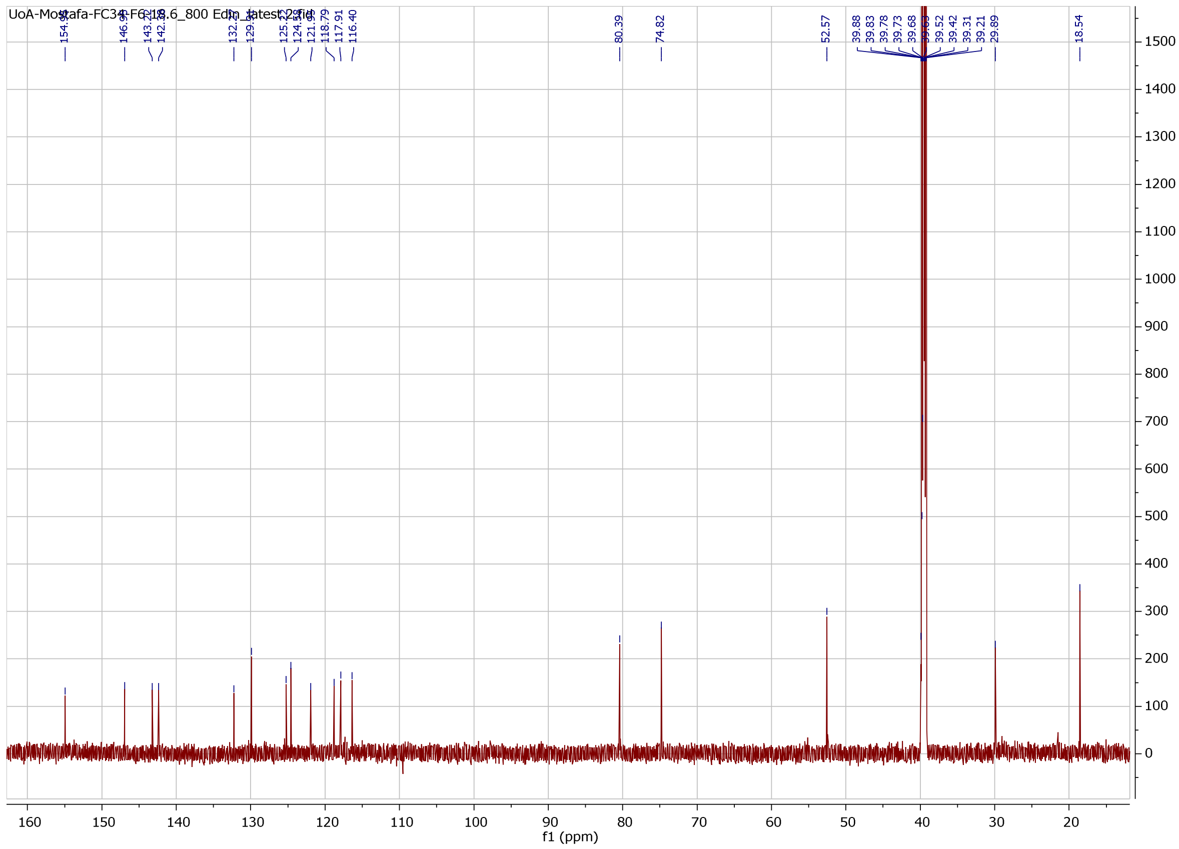

S7. ^{13}C NMR spectrum of luteoride D **2**.

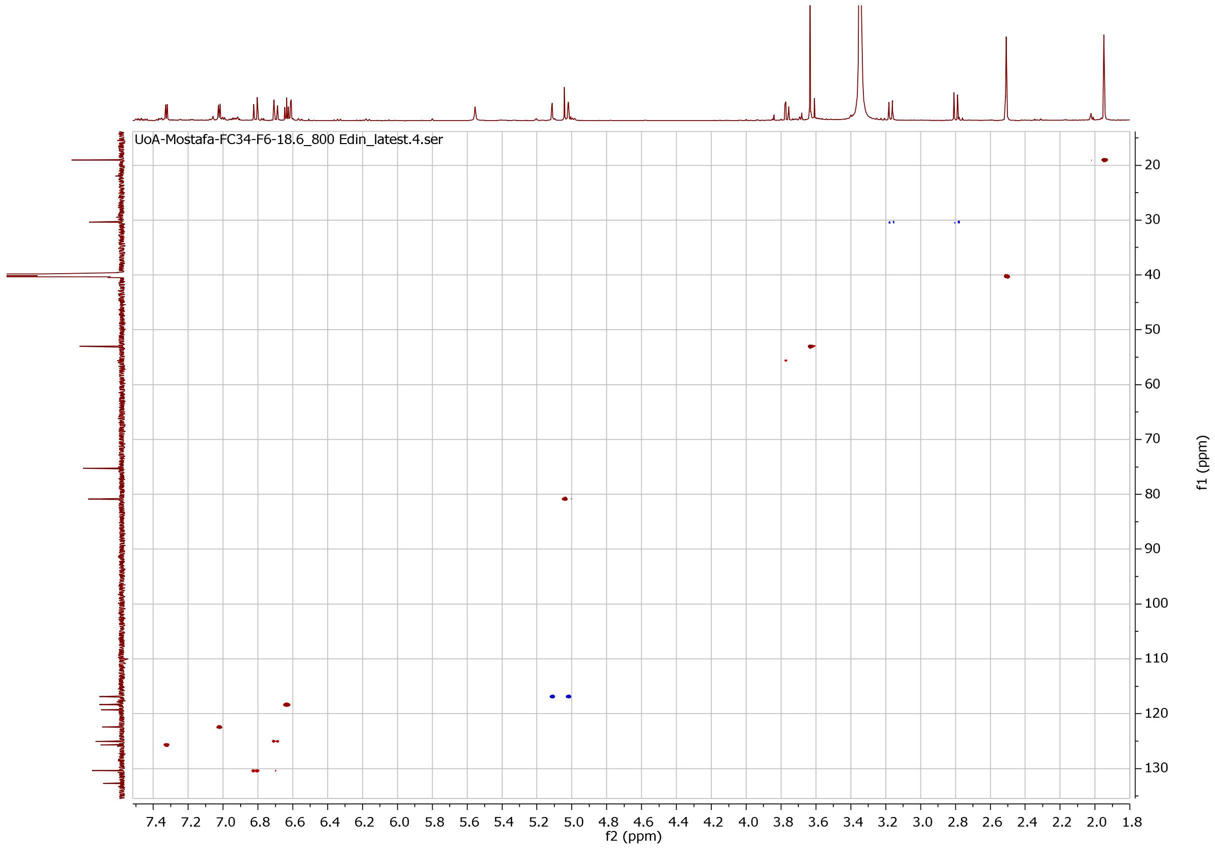

S8. HSQC spectrum of luteoride D **2**.

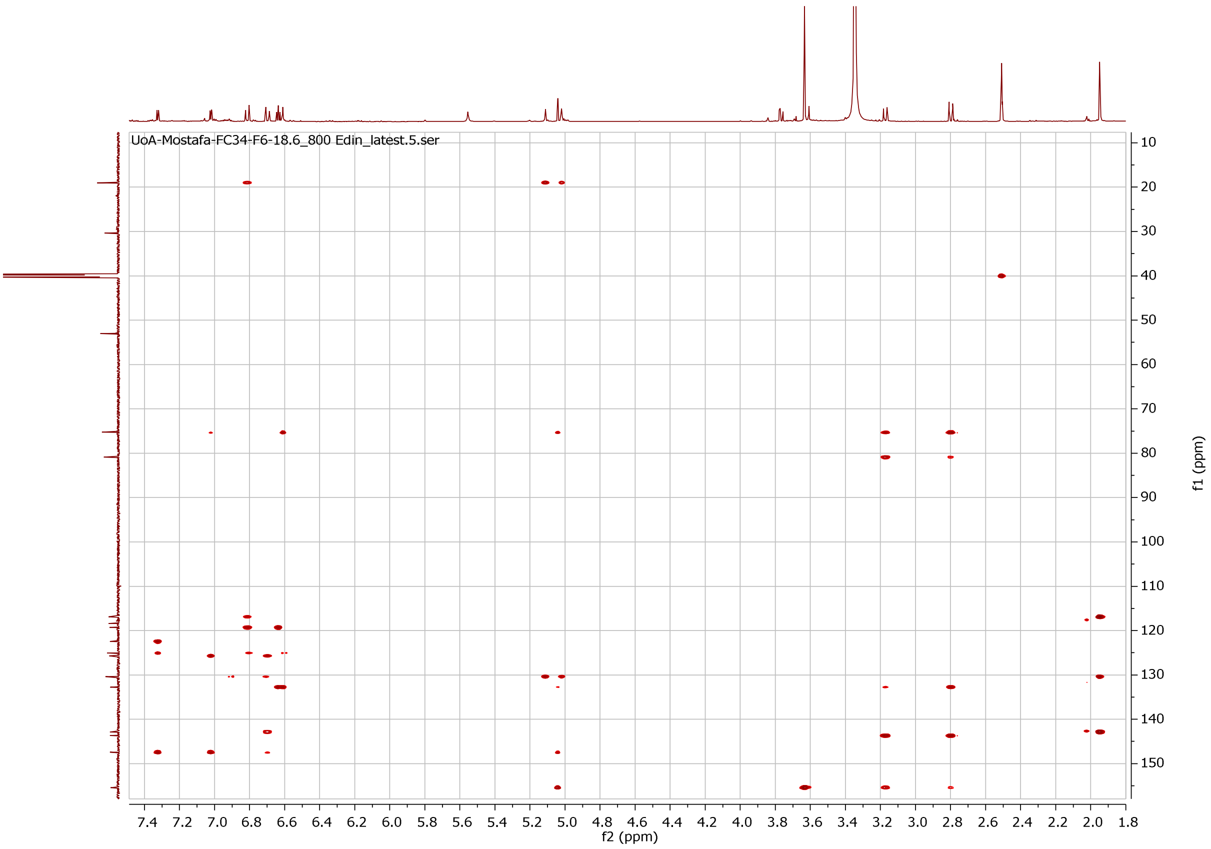

S9. HMBC spectrum of luteoride D **2**.

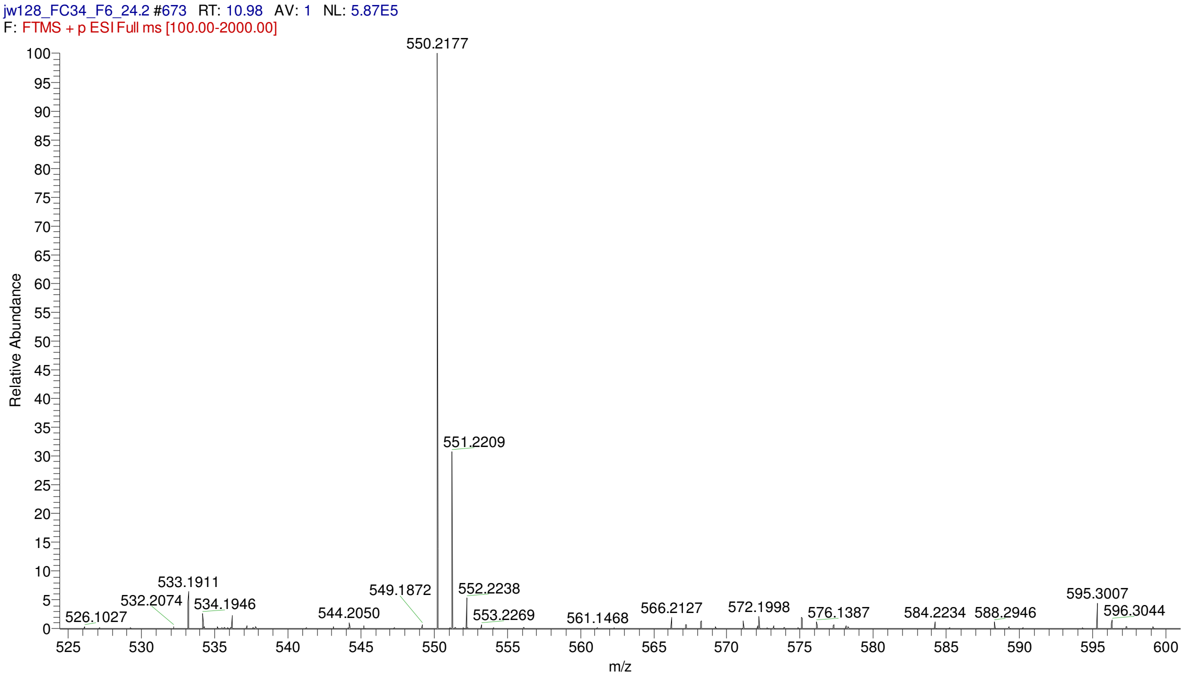

S10. HRESIMS analysis of pseurotin G **3**.

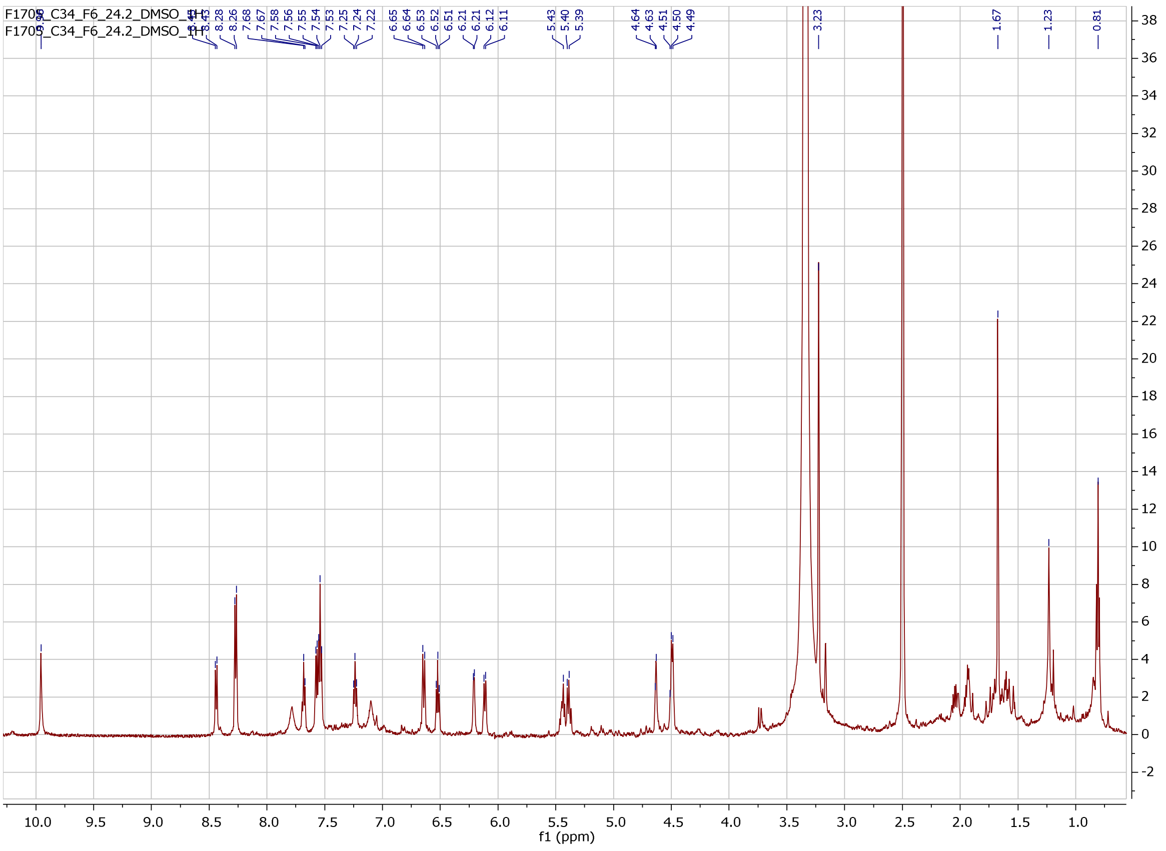

S11. ^{1}H NMR spectrum of pseurotin G **3**.

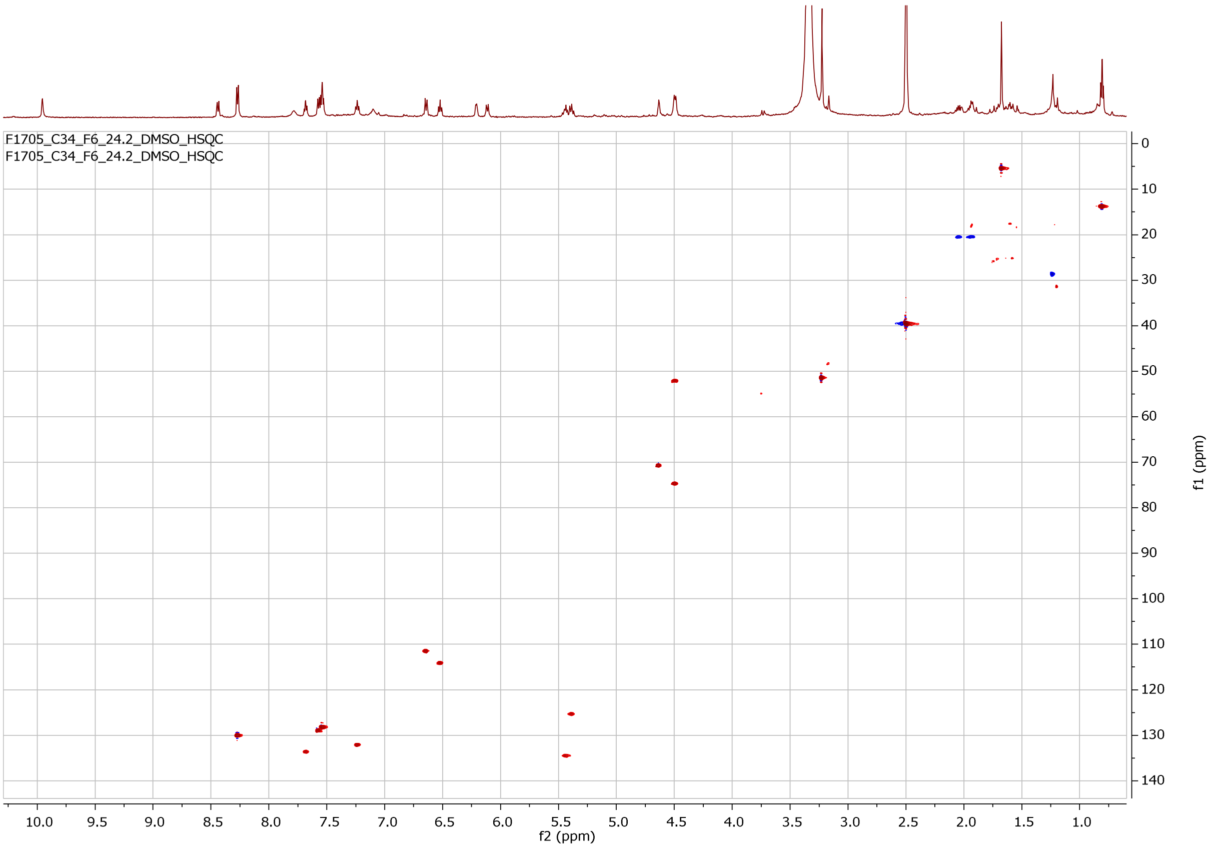

S12. HSQC spectrum of pseurotin G **3**.

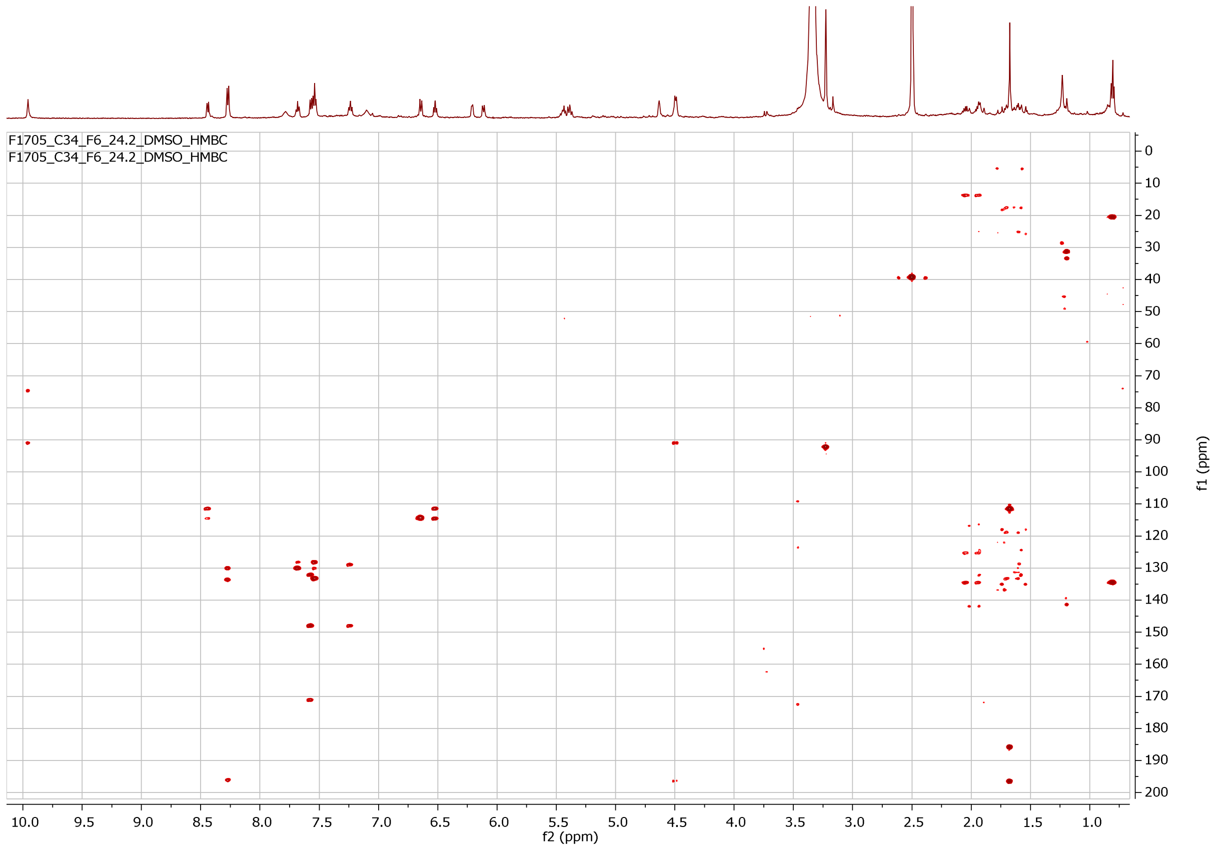

S13. HMBC spectrum of pseurotin G **3**.

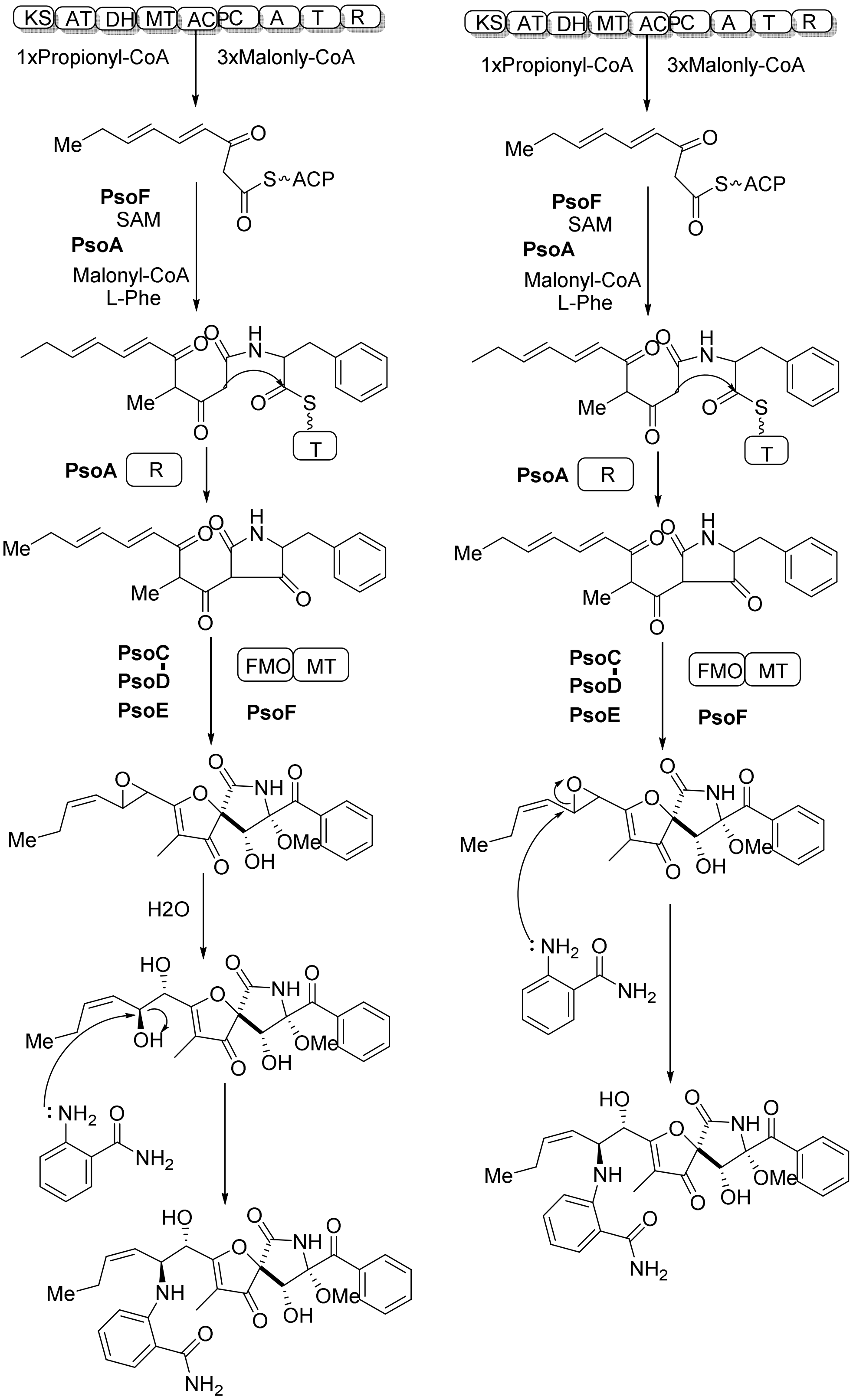

S14. Proposed biosynthetic pathway of pseurotin G **3**.
